# Supplementary material for: Conservation genomics of urban populations of Streamside Salamander (Ambystoma barbouri)
Source: PLoS One. 2022 Jun 30;17(6):e0260178. doi: 10.1371/journal.pone.0260178 (PMC9246143; doi:10.1371/journal.pone.0260178)
Supplement: S1 Table — (DOCX) [file pone.0260178.s001.docx]

**Supplement 1.** GenBank accession numbers for mitochondrial D-loop sequences used in phylogenetic reconstructions.

| **Population ID (Map ID)** | **Genbank Accessions** |
| --- | --- |
| Bedford 6 | OM54086-OM540867 |
| Davidson 3 | OM540868-OM540870 |
| Rutherford 1 | OM540871- OM540873 |
| Rutherford 7 | OM540874- OM540885 |
| Rutherford 9 | OM540886- OM540888 |
| Sumner 2 | OM540889- OM540891 |
| Sumner 5 | OM540892- OM540894 |
| Sumner 7 | OM540895- OM540899 |
| Sumner 8 | OM540900- OM540905 |
| Wilson 1 | OM540906- OM540910 |
| Wilson 3 | OM540911- OM540874 |
| Wilson 4 | OM540915- OM540917 |
| Williamson 2 | OM540918- OM540920 |
| *A. barbouri -* Kentucky  Kentucky FC | OM540846- OM540848 |
| Kentucky RR | OM540851- OM540855 |
| Kentucky SL | OM540856- OM540859 |
| Kentucky SW | OM540860- OM540863 |
| Outgroups |  |
| *A. mabeei* | OM540921- OM540922 |
| *A. maculatum* | OM540923- OM540924 |
| *A. talpoideum* | OM540925 |
| *A. texanum* | OM540926 |
