# Supplementary material for: Conservation genomics of urban populations of Streamside Salamander (Ambystoma barbouri)
Source: PLoS One. 2022 Jun 30;17(6):e0260178. doi: 10.1371/journal.pone.0260178 (PMC9246143; doi:10.1371/journal.pone.0260178)

**Supplement 2.** Assignment probabilities from DAPC analysis for A.) sampled populations of *A. barbouri* in Tennessee B.) sampled populations of *A. barbouri* in Tennessee and Kentucky, and C.) all sampled populations of *A. barbouri* and two representative populations of *A. texanum*.
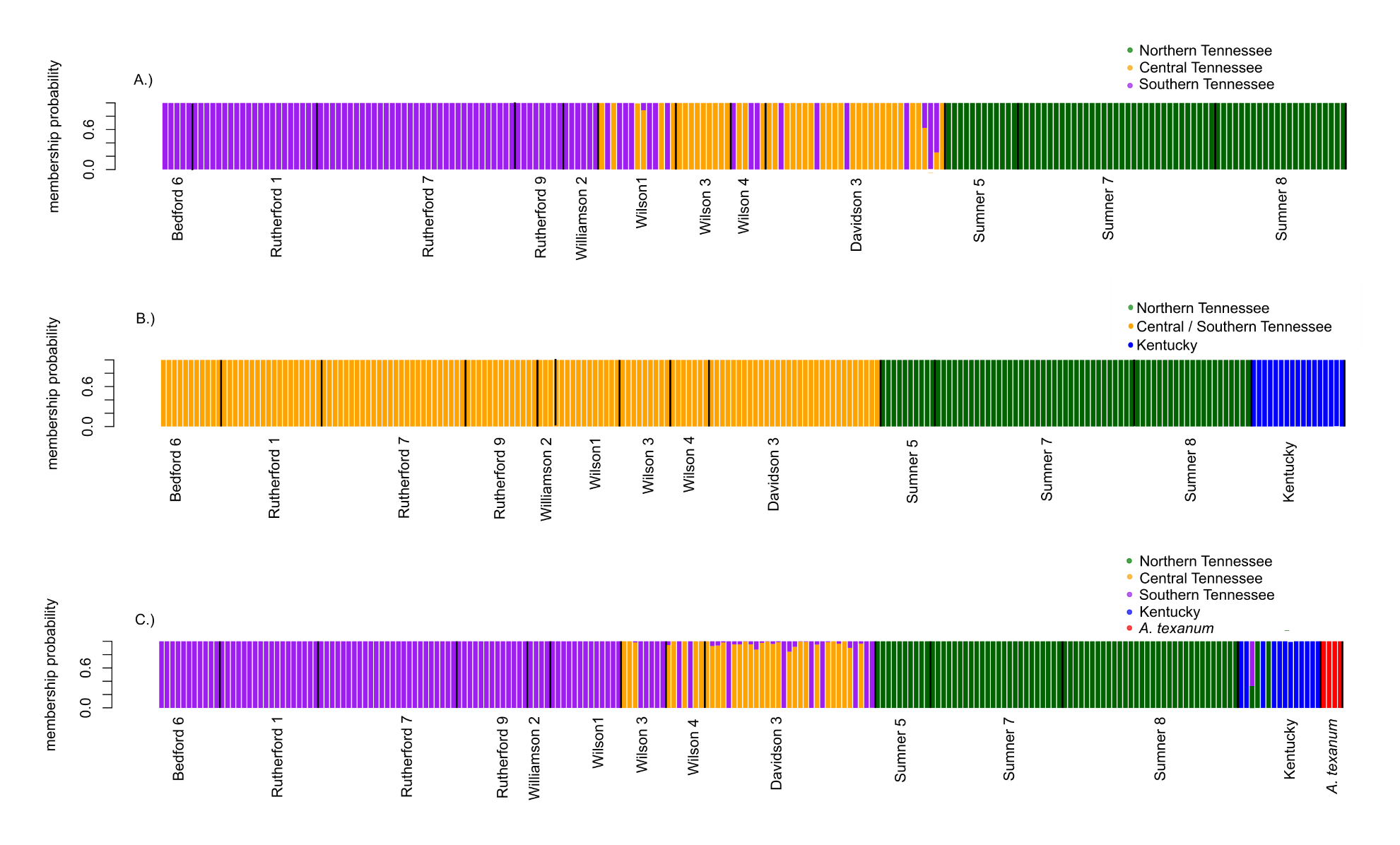

Supplement: S1 Fig — Assignment probabilities from DAPC analysis for A.) sampled populations of A. barbouri in Tennessee B.) sampled populations of A. barbouri in Tennessee and Kentucky, and C.) all sampled populations of A. barbouri and two representative populations of A. texanum. (DOCX) [file pone.0260178.s002.docx]
